# Egg-mediated maternal effects in a cooperatively breeding cichlid fish

**DOI:** 10.1038/s41598-023-35550-5

**Published:** 2023-06-16

**Authors:** Maria Reyes-Contreras, Bonnie de Vries, J. C. van der Molen, T. G. G. Groothuis, Barbara Taborsky

**Affiliations:** 1 Division of Behavioural Ecology, Institute of Ecology and Evolution, University of Bern, Wohlenstrasse 50A, 3032 Hinterkappelen, Switzerland; 2 The Groningen Institute for Evolutionary Life Science, University of Groningen, Nijenborgh 7, 9747 AG Groningen, The Netherlands; 3 Laboratorium Bijzondere Chemie, Cluster Endocrinologie and Metabole Ziekten, University Medical Center Groningen, 9700 RB Groningen, The Netherlands

**Keywords:** Evolutionary ecology, Evolution, Physiology

## Abstract

Mothers can influence offspring phenotype through egg-mediated maternal effects, which can be influenced by cues mothers obtain from their environment during offspring production. Developing embryos use these components but have mechanisms to alter maternal signals. Here we aimed to understand the role of mothers and embryos in how maternal effects might shape offspring social phenotype. In the cooperatively breeding fish *Neolamprologus pulcher* different social phenotypes develop in large and small social groups differing in predation risk and social complexity. We manipulated the maternal social environment of *N. pulcher* females during egg laying by allocating them either to a small or a large social group. We compared egg mass and clutch size and the concentration of corticosteroid metabolites between social environments, and between fertilized and unfertilized eggs to investigate how embryos deal with maternal signalling. Mothers in small groups produced larger clutches but neither laid smaller eggs nor bestowed eggs differently with corticosteroids. Fertilized eggs scored lower on a principal component representing three corticosteroid metabolites, namely 11-deoxycortisol, cortisone, and 11-deoxycorticosterone. We did not detect egg-mediated maternal effects induced by the maternal social environment. We discuss that divergent social phenotypes induced by different group sizes may be triggered by own offspring experience.

## Introduction

Egg-mediated maternal effects, where mothers influence the size or composition of eggs, can shape offspring phenotype. These effects are taxonomically widespread (insects^1^, birds^2^, fish^3^, amphibians^4^, and reptiles^5^). These maternal effects can occur, for instance, by the adjustment of egg mass^6^, which is often considered as proxy for the amount of nutrients present in eggs such as lipids and proteins (e.g., in fish eggs^7^), or by adding hormones^2^, and antioxidants (carotenoids, vitamins) as shown in bird eggs^8^. Egg-mediated maternal effects can be adaptive for offspring, for mothers, or both. They can be adaptive for offspring when maternal effects provide information enabling offspring to adjust their phenotype to the predicted conditions encountered after birth^9^; if this enhances maternal reproductive success, maternal and offspring fitness optima are aligned. They may also be adaptive only for mothers, at the cost for offspring fitness, if they allow mothers to reduce the investment per single offspring and to have either more offspring in the current brood or higher survival and reproductive success in the future. The latter maternal effects may result in parent–offspring conflict and offspring evolving resistance to maternal effects^9^.

Mothers can influence offspring lifetime fitness by enhancing their investment in egg quality. For example, egg mass typically correlates positively with offspring size after birth (rev. in^10^). Larger offspring often have better survival prospects because they are more mobile^11^ and are more likely to survive the earliest life stages^12,13^, if their main predators are gape-size limited^14^. Eggs can also be endowed with particular nutrients enhancing predator evasion. For instance, in a teleost fish, the red drum (*Sciaenops ocellatus*), a higher content of certain unsaturated essential fatty acids in their eggs resulted in larvae with faster escape responses^15^. In birds, egg mass was positively correlated with offspring lifetime fitness^16^. A higher investment in the offspring quality may have to be traded for quantity, as documented for instance in anseriform birds^17^. If mothers solve this trade-off in favour of higher offspring numbers, parent–offspring conflict is expected to arise.

Alternatively to clutch size and egg nutrient allocation, mothers can also use hormone signalling via modification of egg composition to shape offspring phenotype in various ways^18^, including their later behaviour^19^. Hormones are known to have long term organizational effects during early embryonic developmental periods. Maternal hormone signalling plays an important role in shaping the hormonal environment of developing embryos^18,20–22^. It is influenced by environmental cues perceived by mothers, such as the nutritional quality of the environment, risk, or social stability, which in turn can influence offspring growth, gene expression, and behaviour^18,23–25^. In mammals, embryos can be influenced by maternal hormones via the placental blood stream during the entire gestation period^26^. In contrast, in oviparous species, the maternal influence on the embryonal hormonal endowment is restricted to the egg formation period, which allows studying maternal and embryonal influences on offspring phenotype separately. This separation also allows developing embryos to modulate the maternal hormonal signals independently from maternal influence in case there is a conflict between the interests of mothers and offspring.

In highly social species like cooperative breeders, communal breeders and eusocial species, the social environment is key in shaping morphology^27^, physiology^1^, and behaviour^28^. The early social experience (e.g.,^29,30^) and maternal effects can both shape the social phenotype of offspring^1^. In cooperative breeders, conflict between parents and offspring may arise over offspring dispersal tendencies, if parents need help at their territory, while offspring might prefer to breed independently. For instance, the dispersal propensity in cooperative breeders can be influenced by early social experience such as the experienced group composition^30^.

A key candidate mechanism for modulating social phenotype in dependence of maternal and early social cues is the activity of the vertebrate ‘stress axis’ (i.e., the hypothalamic–pituitary–adrenal/interrenal axis^25,31^) with glucocorticoid (GC) hormones as major signalling hormones^32^. GCs and glucocorticoid receptors (GRs) facilitate social behaviour of vertebrates in multiple ways^28^. GCs favour the propensity to show alloparental care in wild vertebrates^28^; in social cichlids, being reared in a socially more complex environment leads to an increase in GR mRNA expression in the brain as well as a higher ability to express appropriate social responses (i.e., higher social competence^33^) during contests^34^ and an enhanced tendency to engage socially with conspecifics^35^. Finally, in laboratory rats, GR expression is involved in the transgenerational, non-genetic transmission of stress axis reactivity, which is mediated by the intensity of tactile contact between mothers and pups^36,37^. Glucocorticoid metabolites can have organizational effects during early development^22^. However, it is unknown whether mothers of cooperative breeders allocate GCs differentially depending on their need of help to influence offspring social and dispersive phenotype, and in case of a parent–offspring conflict over dispersal, whether embryos are able to eliminate them during their development (e.g., see^38^ for a mechanism of teleost embryos to eliminate maternal GCs).

Thus, to understand how maternal effects ultimately shape offspring social phenotype in cooperative breeders, the role of mothers—endowing eggs with different hormones—and of embryos using them needs to be experimentally disentangled. The first step to this end is to compare egg content by mothers in relatively high (small groups) versus low (large groups) need of help and without versus with embryonic development ongoing. A second step would then be to experimentally manipulate the hormones in the eggs to test for causal relationships in the offspring. Here we addressed the first step by experimentally varying the group size of mothers of the cooperatively-breeding cichlid *Neolamprologus pulcher* to elicit environment-induced maternal effects in the eggs. Next, we compared egg mass and clutch size between the two social environments, and we compared the composition of GC metabolites without embryonic development (unfertilized eggs) with GCs present in developing eggs in the two social environments in order to get an indication of whether embryos may eliminate maternal GCs after fertilization. We predicted that females reproducing in small groups lay larger eggs than in large groups^39^, as they have fewer helpers assisting them in defending the offspring against predators. Larger young are more mobile^11^ and have an advantage under size-dependent predation risk as it is common in aquatic environments^14^. Furthermore, we predicted that in small groups, in which there is a higher predation risk and a higher need of help (compared to large groups), females use egg-mediated maternal effects, such as an increased GC deposition. The latter is known to affect offspring behaviour, including an increased fear response to predators and potentially to reduce explorative behaviour^40^, which in turn could decrease offspring propensity to disperse. Because of the higher predation risk in small groups of cooperative breeders^41–45^, the incentives to disperse should be higher in small-group offspring. Therefore, we predicted the potential for parent–offspring conflict over dispersal to be higher in small groups. Thus we expected embryos from small groups to have a higher likelihood to eliminate maternal GCs than embryos in large groups.

## Results

### Egg mass and clutch size

**Figure 1:**
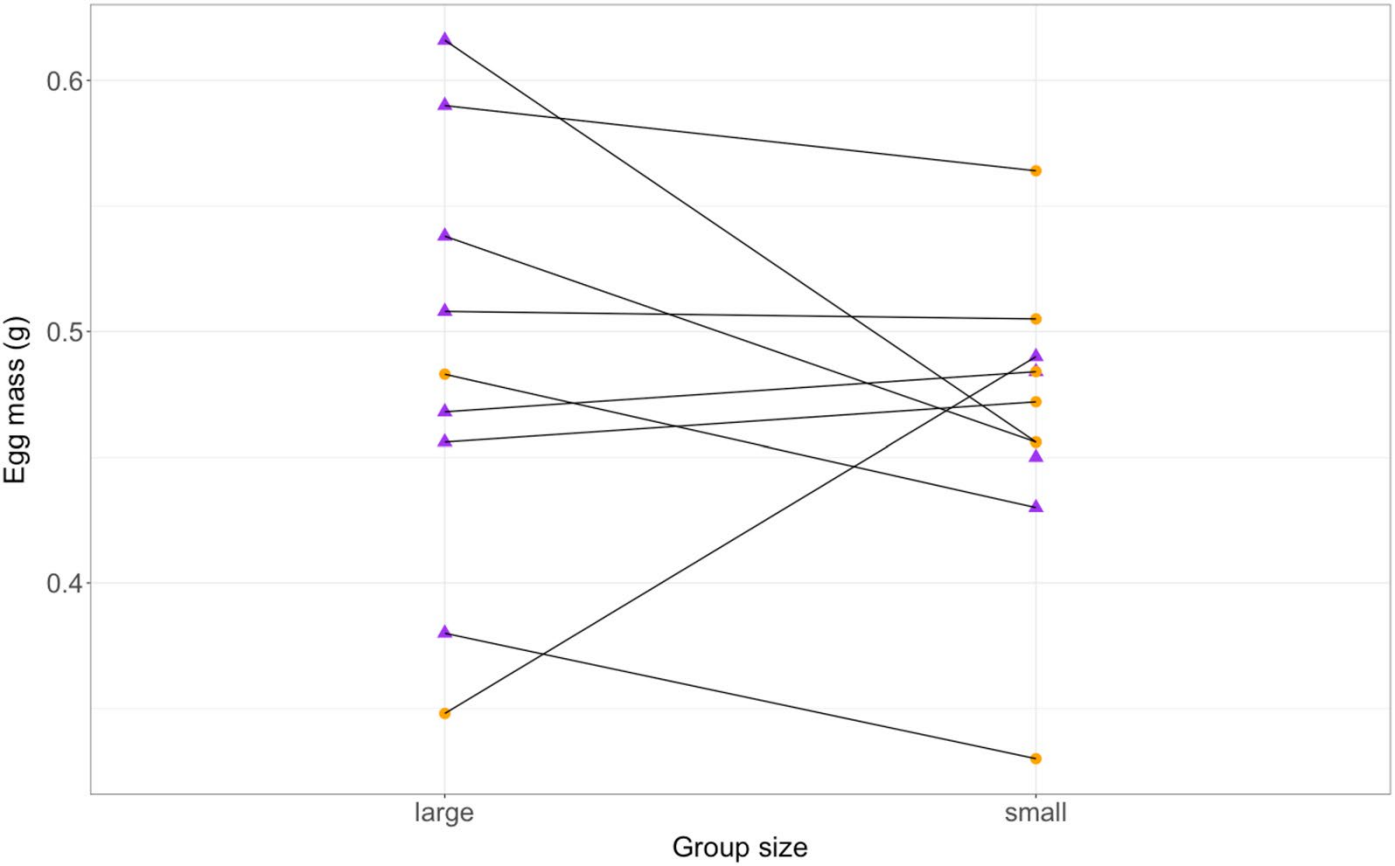
Egg mass of individual females under the two different group size conditions. The clutches of spawning 1 are represented by orange circles and the ones from spawning 2 by purple triangles.

Egg dry weight did not differ between females reproducing in large and small groups (d = 0.20; c.i. = − 0.45 to 0.85, Table 1; full model in Table S2), and spawning sequence (i.e., spawning 1 and 2) did not significantly affect egg mass (d = 0.50, c.i. = − 0.11 to 1.08) (Fig. 1). Female body condition tended to affect egg mass (Table 1).

**Table 1:** Results of the final two models, first linear mixed model (LMM) to test the effect of group size, spawning sequence (i.e., spawning 1 or 2), and female body condition on egg mass.

	Estimate ± S.E	t	z	*p*
*Egg mass*				
Intercept	0.0003 ± 0.00009	3.23		0.0089
Spawning sequence (spawning 2)	0.00005 ± 0.00003	1.73		0.11
Group size (small)	0.00002 ± 0.00003	0.61		0.56
Female body condition	0.00005 ± 0.00003	1.86		0.088
*Clutch size*				
Intercept	2.3531 ± 0.5968		3.943	< 0.001
Spawning sequence (spawning 2)	0.57 ± 0.175		3.25	**0.0012**
Group size (small)	0.348 ± 0.174		2	**0.045**
Female body condition	0.54 ± 0.179		3.02	**0.0025**

Sample size: small groups n = 11 clutches (spawning 1: n = 7, spawning 2: n = 4); large groups: n = 9 clutches, (spawning 1: n = 2, spawning 2: n = 7). Second the generalized linear mixed-effect model (GLMM) to test the effect of group size, spawning sequence (i.e., spawning 1 or 2), and female body condition on clutch size. Sample size: small groups n = 10 clutches (spawning 1: n = 7, spawning 2: n = 3); large groups: n = 11 clutches, (spawning 1: n = 4, spawning 2: n = 7). Estimates refer to the factor levels given in brackets. Significant *p*-values are in bold (except for the intercept).

**Figure 2:**
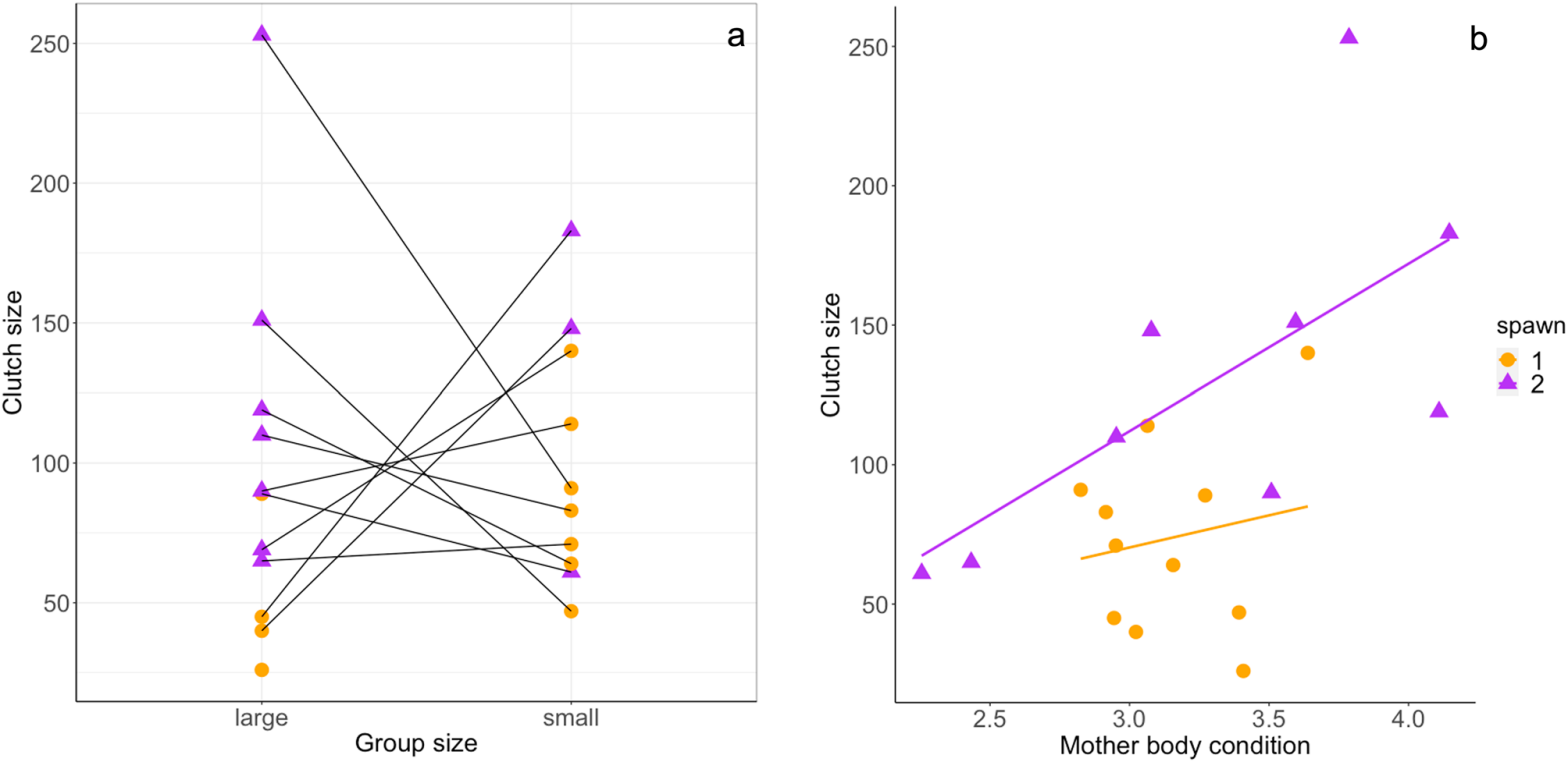
(**a**) Clutch sizes in the different group size treatments. The clutches of spawning 1 are represented by orange circles and the ones from spawning 2 by purple triangles. (**b**) Clutch size as a function of mother body condition. The body condition was calculated using Fulton’s index.

The interaction between group size and spawning sequence did not significantly influence clutch size (d = 0.34; c.i. = − 0.10 to 0.78, see full model in Table S3). Females in small groups laid significantly more eggs (d = 0.59; c.i. = 0.15 to 1.03, Table 1) and females in the second spawning laid significantly larger clutches irrespective of female body condition (d = 0.80; c.i. = 0.36 to 1.24, Table 1). In addition, female body condition positively affected clutch size (d = 0.68; c.i. = 0.24 to 1.12; Fig. 2a,b, Table 1).

Finally, we tested whether egg mass and clutch size were negatively correlated, as expected in case of a trade-off between these traits, but this was not the case (Pearson correlation, r = − 0.042, *p* = 0.86).

### Egg corticosteroid concentration

In the PCA of corticosteroid concentrations in the eggs, PC1 explained 64.96% of the variation, and PC2 explained 26.87% while the other two PCs together explained only 8.17% of the variance (Table S4). We further analysed the individual scores of PC1 and PC2. Three metabolites explain a similar amount of the variance explained by PC1, 11-deoxycortisol (34.85%), 11-deoxycorticosterone (32.45%), and cortisone (27.33%). Instead, the cortisol-like compound explains 78.29% of variance explained by PC2 (Table 2).

**Table 2:** Contribution of each steroid hormone to the first and second dimension of the PCA (PC1 and PC2) of maternal steroid allocation to unfertilized and fertilized eggs laid in large and small groups.

Dimensions	Variable	Variance explained in %
PC1	11-Deoxycortisol	34.85
	Cortisone	27.33
	11-Deoxycorticosterone	32.45
	Cortisol-like	5.37
*Total of the variance explained*		*64.96%*
PC2	11-Deoxycortisol	1.91
	Cortisone	18.66
	11-Deoxycorticosterone	1.14
	Cortisol-like	78.29
*Total of the variance explained*		*26.87%*

**Figure 3:**
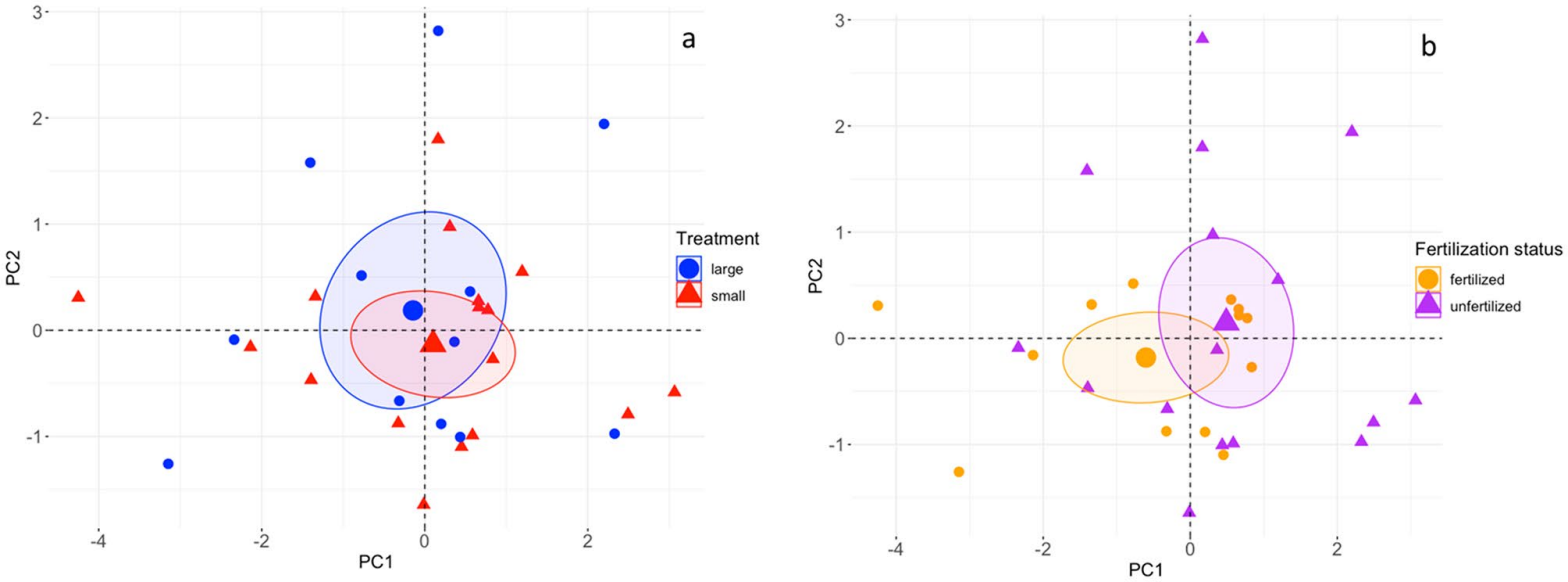
PCA of the four corticosteroid metabolites identified the fish eggs for samples of both group sizes and fertilization states. (**a**) Large (blue circles) and small (red triangles) groups. (**b**) Fertilized (orange circles) and unfertilized (purple triangles) eggs. In both panels, individual samples are depicted with small symbols whereas the mean value of each group size is depicted with large symbols.

Next, we analysed the individual scores of the PCA for effects of group size and fertilization status. Group size did not affect the PC1 scores, (d = 0.08; c.i. = − 0.31 to 0.46; Fig. 3a) and corticosteroid metabolites between unfertilized and fertilized eggs for PC1 did just not reach statistical significance (d = 0.39, c.i. = − 0.02 to 0.78; Fig. 3b see Table S5 for full initial model, final model Table 3). Because three of the four metabolites explained a similarly high percentage of variation in PC1 (11-deoxycortisol, 11-deoxycorticosterone, and cortisone, in total 94.63%), and all three load positively on PC1 (Fig. S2), this result show that less of these hormones tended to be present in fertilized than in unfertilized eggs. For PC2, steroid metabolite composition did not significantly differ between group size treatments or fertilization status (effect size: group sized d = 0.16, c.i. = − 0.24 to 0.55; fertilization status d = 0.14; c.i. = − 0.26 to 0.53; LM, group size treatment: estimates ± s.e. = − 0.265 ± 0.412, t = − 0.642, *p* = 0.50; fertilization status: estimates ± s.e. = 0.277 ± 0.408, t = 0.678, *p* = 0.48, see Table S6 for full initial model).

**Table 3:** Summary table of linear model that tested the effect of group size and fertilization status on the PC1 scores.

	Estimate ± S.E	t	*p*
Intercept	− 0.934 ± 0.609	− 1.53	0.14
Group size (small)	0.478 ± 0.609	0.79	0.44
Fertilization status (unfertilized)	1.184 ± 0.603	1.96	0.06

## Discussion

In this study, we asked whether social group size, determined by the number of brood care helpers present in a group, and the fertilization state influence egg traits of females that may influence helper behaviour of the offspring in the cooperatively breeding cichlid *N. pulcher*. We had hypothesized that females with few helpers (i.e., in small groups) produce larger offspring, which have a survival advantage, and may use egg-mediated maternal effects to decrease offspring probability to disperse from the natal territory. In brief, the size of social groups, the spawning sequence, and female body condition significantly influenced clutch size. Females with fewer helpers laid larger clutches than females in large groups. Females that were re-allocated to a second social group laid larger clutches in comparison with females in the first social group. Females with a higher body condition laid larger clutches irrespective of the group size. Contrary to clutch size, group size did not influence egg mass, and there was no indication of a trade-off between clutch size and egg mass. The concentration of the yolk corticosteroid metabolites 11-deoxycortisol, 11-deoxycorticosterone, and cortisone all loaded on the same principal component and tended to be lower in fertilized eggs in comparison with unfertilized egg, although it did just not reach statistical significance. In addition, there was an unknown corticosteroid metabolite present in eggs, which we identified as a 11-deoxy metabolite. It lacks hydrogens in the 11 positions, and it is 95% similar to cortisol.

A possible explanation for females producing larger clutches when being in small groups is the lower survival prospects for juvenile and adult group members in small groups^41–44^. Females in small groups may produce more offspring to ensure the survival of at least some offspring because of these lowered survival prospects. At the same time, the need for more helpers to grow up and to join in brood care at the juvenile stage is higher in small than in large groups^45,46^, which also should favour larger clutches to be produced by females in small groups. Finally, offspring of small groups may have a higher propensity to disperse later in life because of the higher mortality risk prevalent in these groups. Thus, lower survival prospects in the natural environment, a higher need for more helpers and possibly a higher dispersal tendency away from small groups together may explain larger clutch sizes laid in small groups.

Females had larger clutches also in the second spawning of the spawning sequence. It is possible that there is a positive effect of female age on reproductive traits. For example, six-month-old zebra finches (*Taeniopygia guttata*) female produced larger clutches in comparison with three-month-old females^47^ and in female Artic charr (*Salvelinus alpinus*) egg mass is positive correlated with female age^48^. *N. pulcher* females can produce a new clutch every 15–30 days and we collected four clutches in each spawning sequence. Hence, at the second spawning (i.e., in the second social group) females were at least two months older than at the first spawning, which might explain the production of larger clutches in the second spawning, irrespective of female body condition, which was statistically controlled for.

In addition, clutch size increased with female body condition regardless of group size and spawning sequence. This positive relationship has been previously reported for threespine stickleback fish (*Gasterosteus aculeatus*)^49^. It seems plausible that *N. pulcher* females with better body condition laid larger clutches because they are able to divert more of their energetic resources into egg production.

In an earlier study, *N. pulcher* females were shown to lay smaller eggs in the presence of more helpers, presumably allowing females to save energy for the next reproductive event^39^. More recently, a meta-analysis^50^ confirmed that in cooperatively breeding fish and birds there is a general tendency of breeder females to reduce egg mass if they received more help, suggesting load-lightening by a higher number of helpers. To the contrary, in our study mothers adjusted clutch size instead of egg mass to group size, with no evidence for a trade-off between clutch size and egg mass (see absence of trade-off also in previous work in *N. pulcher*^39^ and in lizards^51^). Possibly females are unable to plastically adjust egg size^49^ in response to short-term changes of group size. Although females were able to lay at least four clutches in each group size treatment, longer-term measurements of more reproductive events may be necessary to detect egg mass adjustment to the size of the social group. Alternatively, dominant breeder females may use other mechanisms different from egg mass to increase offspring probability to remain philopatric and become broodcare helpers, such as varying the egg composition by provision offspring with differential concentrations of proteins, lipids, vitamins, hormones, or maternal transcripts to shape offspring phenotype.

The physiological mechanisms underlying adaptive maternal effects are often cryptic and therefore poorly understood^52^. There is ample evidence, however, that maternal effects can be mediated by hormones deposited in eggs^2,53,54^ or, in mammals, they can be transmitted to the embryos by the maternal blood stream^55,56^. During reproduction of oviparous species, maternal hormones have a dual function: in (i) promoting offspring developmental processes^57^ and (ii) fine tuning physiological functions in the maternal body during the reproductive phase^2,58^. The corticosteroid hormonal profiles of mothers and of the hormones deposited in eggs are often correlated. In fish, maternal circulating cortisol enters the vitellogenic follicle either via diffusion or by binding to yolk proteins, which suggests that a high corticosteroid hormone concentration in maternal plasma due to stressors may spill over to the embryo and may generate long-term effects on embryo phenotypic traits^59^.

The corticosteroid metabolites detected in fertilized and unfertilized eggs of our study may have a relevant biological function before and after fertilization. The metabolite 11-deoxycortisol is a maturation-inducing steroid of teleost oocytes^60^. Cortisone can be converted to cortisol in the last weeks of human fetal development^61^, but teleost embryos lack the enzyme 11β-hydroxysteroid dehydrogenases type 1, which metabolizes cortisone to cortisol^62^; hence, it is difficult to hypothesize about a biological function of cortisone in teleost eggs. The metabolite 11-deoxycorticosterone is a ligand for the mineralocorticoid receptor (MR) in teleost fish^63^. In zebrafish, maternal RNA expression of mineralocorticoid receptor (*mr* RNA) has a low abundance, but after twelve hours of fertilization it increases, which makes MRs available for binding to 11-deoxycorticosterone^64^. Additionally, it has been suggested that the 11-deoxycorticosterone-MR axis may be involved in the early developmental process and regulation of development after hatching in teleosts^65^. In contrast to previous teleost egg analyses^66–68^, in our corticosteroid profiles, cortisol was not detected. Possibly cortisol was converted to ‘cortisone’, as it has been reported from unfertilized oocytes of tilapia cichlids (*Oreochromis mossambicus*). In these fish, radio labelled cortisol is completely converted to cortisone once it enters the oocytes. The function of cortisone in fish is poorly understood, but it is considered to be an inactive form of cortisol, with no or little affinity to corticosteroid receptors^63^.This means that if cortisol diffuses into the oocyte from the maternal circulation^59^, it is converted to cortisone while the oocyte is still inside the mother and once it is ovulated it lacks cortisol. This may explain the absence of cortisol in unfertilized and fertilized eggs in our experiment. As a fourth corticosteroid, we detected an unknown metabolite that was by 95% similar to cortisol and the function of which is not known.

Contrary to our predictions, we did not detect differences between corticosteroid concentrations in eggs produced by females in different group sizes. It is possible that a difference in group size per se does not represent a strong enough stressor leading to an increase circulating maternal corticosteroid concentration that will differentially affect the oocytes. Alternatively, even if maternal corticosteroid concentrations were high enough to spill over to oocytes, the lack of differences between social groups may be explained by the fact that teleost oocytes, which are inside the female ovarian follicle^60^, are protected against high levels of maternal circulation levels of cortisol. This is because high levels initiate the transcription of the enzyme 11β-hydroxysteroid dehydrogenase type 2 in the theca and granulosa cells, which are monolayers of cells that surrounds the oocyte^60^ being responsible for the conversion of cortisol to cortisone^69^.

Analysing the individual scores of our PCA on corticosteroid metabolites revealed that the three corticosteroid metabolites loading on PC1 were present in lower amounts in fertilized eggs than in unfertilized eggs (Fig. 3b), even if the difference just not reached statistical difference. This marginal difference of corticosteroid concentration between fertilized and unfertilized eggs may have several possible explanations. First, it can be attributed to metabolization of the three corticosteroids after fertilization^70^. Embryos inside an egg may have converted maternally deposited corticosteroids. For example, chicken embryos convert glucocorticoid hormones to 20-β-dihydrocortisol^71^, which requires a set of enzymes^2^. In zebrafish, cortisol is metabolized to cortisone, which is further metabolized to 20β-hydroxycortisone, and the latter is excreted^72,73^. Second, the metabolites can be converted to another corticosteroid molecule before fertilization^71,74^. Third, they can be excreted from the oocyte before^74^ and after fertilization^38^. If embryos were playing an active role in eliminating GCs from eggs because of parent–offspring conflict over offspring dispersal, we had expected to find a stronger GC reduction in eggs produced in small groups, which incur a higher mortality risk in the wild^42^. Contrary to this prediction, the reduction of GCs after fertilization did not differ between embryos from small and large groups. Therefore we cannot speculate about a possible function of the GC reduction in offspring resistance to maternal GC signalling.

In summary, while we found an effect of group size during reproduction on clutch size, we do not have evidence that the social environment induces egg mass differences or corticosteroid-mediated maternal effects to shape offspring phenotype. The social environment of *N. pulcher* females may induce other egg-mediated maternal effects such as endowment with vitamins^8^, other hormones^22^ or maternal transcripts^3^. Alternatively or in addition, in our study species, the social environment shapes offspring phenotype directly by way of developmental plasticity. In *N. pulcher* offspring social, and helping behaviours as well as dispersal propensity are plastically adjusted to the composition and size of groups they experience early in life^34,75–78^. Theoretical models predict that at least under strong selection offspring plasticity decreases the magnitude of maternal effects because by being plastic offspring can use direct environmental information^79^. Developmental plasticity allows individuals to integrate cues during their development such that their phenotype is adapted to local conditions. Hence, offspring may actively scan their own social environment for informative cues to plastically adjust their phenotype, and either to strive for independent reproduction or to forego reproduction and help raising breeders’ offspring. This may render egg-mediated maternal effects relatively less important. Instead mothers may influence the behaviour of offspring in the juvenile stage, by preventing dispersal or enforcing help^80–83^.

## Conclusions

The social environment can modulate offspring phenotype via maternal effect and offspring own experience. The absence of egg-mediated maternal effects that provide a head-start and may shape offspring stress axis by hormonal endowment suggest that mother and offspring fitness benefits may be aligned during offspring early developmental period. Later in life, the same social environment may shift mother and offspring fitness optima with the possibility of conflict of interest between parties.

## Methods

### Study species

*Neolamprologus pulcher* is a cooperatively-breeding cichlid endemic to Lake Tanganyika, East Africa^84^. The groups consist of a dominant breeding pair and a variable number of subordinate individuals of different sizes and ages^84^, which help to raise the offspring of the current breeding pair (‘brood care helpers’). Helpers can obtain inclusive fitness benefits if they are related to the breeder’s offspring^84^. Moreover, all helpers obtain direct benefits by access to shelters in the breeders’ territory, which is indispensable for survival because predation risk is high^85^. Individual survival^42^ and the persistence of groups over time^45^ increase with group size. Breeding females reduce their egg mass with increasing group size^39^ and increase it under perceived predation risk during egg maturation^7^. Also, the behaviour of fish later in life is shaped by the early social environment (i.e., group size and composition)^30,34,75,76,78^ and perceived predation threat^30^. The latter occurs both by way of egg-mediated maternal effects^7^ and own offspring experience with predator cues^30,86^. Offspring raised in the presence of more adults and perceived a higher predation risk had a better social competence and were more likely to disperse from social groups for independent breeding^30^.

### Ethical statement

The experiments were approved by the Veterinary Office of the Kanton Bern, Switzerland and conducted in the aquarium facilities of the Ethological Station Hasli of the University of Bern, Switzerland, under the licence number BE 93/18. The methods and experiments were performed in accordance with the Swiss Animal Welfare law and followed the ARRIVE guidelines. The fish used to constitute large and small social groups were taken from the breeding laboratory stock of the aquarium, which originally was derived from wild caught fish from the Kasakalawe point population near Mpulungu, Zambia. At the end of the experiments social group members were reintegrated in the breeding stock. Offspring born in the groups were assigned to another experiment (La Loggia et al. in prep).

### Experimental groups and housing conditions

We set up small and large breeding groups by selecting unrelated fish from the stock tanks of our aquarium facility. A small group consisted of one breeding pair and one helper, which corresponds to the minimal natural group size^87^. In the natural environment, most *N. pulcher* groups contain several helpers of different body sizes and ages^88^. Correspondingly, large groups consisted of a breeder pair and eight helpers of different sizes and sexes (see Table S1 in Supplementary Material); see also^77^. Breeding pairs were assigned to breed either first in a small and subsequently in a large group, or other way round, with the order of group size treatments being balanced across tanks.

The breeding groups were housed in 400-L tanks that were divided in two compartments by opaque, water-tight dividers, one small 100-L compartment for small groups (33 × 65 × 50 cm length × depth × height) and one large 300-L compartment (97 × 65 × 50 cm length × depth × height) for large groups. All compartments were equipped with a 2-cm sand layer, one half flowerpot per fish on the tank bottom as shelters and breeding sites, and additional hiding places mounted near the water surface (empty, semi-transparent plastic bottles). In natural territories all group members have their own hiding place, which they defend against other group members^89^. The water temperature was kept at 27 ± 1 °C and the light–dark cycle was 13:11 h with dimmed-light phases of 10 min in between to simulate natural light conditions. All groups were fed commercial adult flake food (JBL Novo Tanganyika®) five days a week and they received fresh food twice per week. Additional TetraMin Baby® powdered flake food was provided when free-swimming fry were present in a tank.

In natural populations, *N. pulcher* breed in colonies, and territories are always established in close vicinity to neighbouring groups^90^. These neighbouring conspecifics, and heterospecific space competitors, opportunistic egg predators (*Telmatochromis vittatus*), and dangerous piscivorous predators (*Lepidiolamprologus elongatus*) frequently intrude natural territories. Hence, breeders and subordinate helpers are constantly defending their territory against the various competitors and predators^85^. The presence of these threats increases the need for help for the dominant breeders and, in turn, raises their readiness to accept helpers^91^. To mimic natural conditions and to elicit helping behaviours by subordinates, which increases their likelihood to be accepted by the dominant breeders^80^, once a week, we exposed all groups to one of the following helping tasks, where the order of presentations was balanced across tanks. (a) Defence against an egg predator, which consisted of presenting one *T. vittatus* inside a transparent tube during 5 min near the centre of the territory^88^. (b) Territory maintenance, which consisted of digging out sand from the shelter(s) used by the dominant breeders for breeding and/or hiding (shelter use by dominants was established directly before the task, and depending on these observations, one or two shelters were filled with sand)^88^. (c) Defence against an unfamiliar conspecific, presented inside a transparent tube for 5 min near the centre of the territory^92^.

### Production of experimental broods

**Figure 4:**
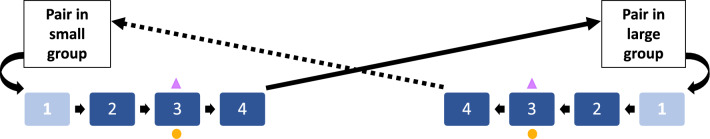
Schematic representation of the egg collection sequence. A breeder pair was assigned either to a small or to a large social group. In each group, breeders produced up to four clutches (i.e., 1,2,3, and 4). The first clutch (light blue box) was removed and discarded. The 2nd and 4th clutches were allowed to hatch and young to grow up in the social groups (dark blue boxes). The 3rd clutch (spawning 1, i.e., orange circle) was either unfertilized or freshly fertilized and collected for analysis for this study. After the 4th clutch, the breeding pair was assigned to a new set of helpers either in a small (dotted arrow) or large (solid arrow) and the eggs of spawning 2 (i.e., purple triangle; unfertilized or freshly fertilized) were collected following same procedure described for spawning 1.

In each group, breeding pairs were allowed to produce at least four clutches (Fig. 4). The 1st, 2nd and 4th clutch were all fertilized and not used for analysis in this study. Only the 3rd clutch generated the samples for this study. It was either unfertilized or freshly fertilized (Table 4).

**Table 4:** Number of independent samples collected to assess clutch size, egg mass, and corticosteroid metabolites in large and small groups, separated by spawning sequence (i.e., laying sequence) and fertilization status.

Measurement	Small group	Large group	Small group	Large group	Small group		Large group	
	Spawning 1 Unfertilized	Spawning 1 Unfertilized	Spawning 2 Unfertilized	Spawning 2 Unfertilized	Fertilized	Unfertilized	Fertilized	Unfertilized
Egg mass	7	2	4	7	–	–	–	–
Clutch size	7	3	3	7	–	–	–	–
Corticosteroid metabolites	–	–	–	–	9	8	4	8

The 1st clutch was removed and discarded; the time to first spawning served to establish new groups and achieve and monitor group stability. Group stability was defined as (i) the absence of evicted individuals, (ii) all group members having access to the bottom of the territory, and (iii) the absence of overtly aggressive interactions between group members. If those criteria were not met before the 1st clutch was laid, the group was re-structured by exchanging members or move them to a different aquarium, which sometimes helps to stabilize groups. The 2nd and 4th clutches were allowed to develop into broods that grew up within their respective group until an age of 2 months and received brood care by all group members (egg cleaning, fanning, guarding). These young were used in a different study (La Loggia MS). The 3rd clutch was collected for analysis of this study (‘spawning 1’).

After producing a 4th clutch, the dominant breeders were moved to another tank where they were merged with a new set of unrelated, unfamiliar subordinate individuals taken from our stock tanks to obtain ‘spawning 2’ (Fig. 4). If a breeder pair had spawned before in a small group, it was now placed in a large group, and, conversely, if it had been in a large group, it was now placed in a small group. Also in this new social group, we collected the 3rd clutch (‘spawning 2’). Hence, spawning 1 and 2 correspond to the laying sequence at which females spawned in a particular social group. We included the spawning sequence in the data analysis, because carry-over effects between clutches may exist, which affect the maternal reproductive strategy in her current social group.

#### Production of unfertilized eggs

We obtained unfertilized eggs to enable us to analyse maternal hormone deposition to eggs, which is unaltered by embryonic development. We prevented fertilization by separating a female ready to lay eggs, further termed ‘gravid female’, from the rest of the group. This was in most cases the dominant breeder female, and in a few cases the large helper female. A gravid female was recognized by her protruded genital papilla and an inflated belly. To collect the eggs of spawning 1 and 2, female reproductive status was checked twice per day for these signs of an approaching spawning. When this occurred, we added one transparent divider to separate the breeder male and the gravid female, and another transparent divider to separate the female from the rest of the group (Fig. S1). Next to the divider that separated the gravid female and dominant male, we placed two adjacent flowerpot halves leaning against each side of the transparent partition such that they formed a “shared shelter” (see Fig. S1). It could be visited by the female and the male simultaneously for spawning, but still prevented physical contact between the breeders so that the sperm released by the male could not reach the eggs^93^. This method has proven successful for collecting unfertilized eggs. Two breeding pairs did not produce an unfertilized clutch in spawning 1.

#### Production of fertilized eggs

If females did not spawn within 10 days, we removed the transparent partition, but we continued monitoring the female and in case a spawning occurred, we collected the fertilized eggs as soon as possible (23.23 ± 5.89 h, mean ± s.e.) after they were laid. Those samples were stored for further analysis of hormonal content to analyse the fate of corticosteroid metabolites after fertilization (see sample sizes for group size treatments and fertilization state in Table 4).

### Sample collection

The unfertilized and freshly fertilized eggs of spawning 1 (i.e., clutch 3 in first social environment) and spawning 2 (i.e., clutch 3 in second social environment) were collected with help of a tweezer, which we used to detach each single egg individually carefully from the surfaces, where we had detected them (e.g., flowerpot, partition, filter). For each clutch we counted the number of eggs.

Furthermore, from all unfertilized clutches, we randomly collected ten eggs and weighed each individual egg to the nearest mg to obtain their fresh weights. Out of those ten eggs, five eggs were randomly selected, dried at 60 °C for 12 h, and then weighed individually to the nearest mg to obtain their dry weight, which was used as proxy of egg mass^94^.

The remaining eggs of each unfertilized clutch as well as all eggs from the fertilized clutches were placed in a cryo pore tubes of 1.6 ml, which were immediately flash frozen in liquid nitrogen, and stored at − 80 °C until corticosteroid extraction. In addition, we measured the length and weight of the female, which had produced the clutch to calculate Fulton’s body condition index, because body condition can influence the number and size of eggs^39^.

### Steroid analysis

#### Background information on teleost steroid pathways

The steroidogenesis pathway in teleost resembles mammalian pathways. It starts by the conversion of cholesterol to pregnenolone. One final metabolite resulting from pregnenolone is 11-deoxycortisol which is further metabolized to cortisol by cholesterol side-chain cleavage enzyme cytochrome P450^63^. Cortisol has been widely reported to be present in teleost eggs in stressed^67,95^ and in non-stressed females^68^. Cortisol can be further metabolized to cortisone^63^. The presence of cortisone has been previously reported in unfertilized eggs of tilapia cichlids (*O. mossambicus*)^74^. Following another path, pregnenolone can be metabolized to 11-deoxycorticosterone and further to corticosterone^63^. The corticosteroid metabolite 11-deoxycorticosterone has not been reported in unfertilized teleost oocytes but is an important regulator of female’s oocyte maturation^96^.

#### Steroid extraction and measurement in eggs

We had used a paired design for clutch collection with the same females laying eggs both in a large and a small group, as we aimed to control for between-female variability in egg mass and clutch size. However, in some cases also large helper females spawned, and importantly many of the clutches were too small to provide enough material for the corticosteroid analysis. Therefore, we had to pool clutches in this analysis including eggs from multiple females within the same group size treatment and the same fertilization state (Table 4) to reach approximately 100 mg per sample. The final mass of the samples was 92.59 ± 5.25 g (mean ± standard deviation).

The frozen eggs of the pooled samples were grinded using a TissueLyserII, weighed and diluted to 600 mg with DPBS (Gibco DPBS (1x); Dulbecco’s Phosphate Buffered Saline; REF14190-094). To each unfrozen sample 75 µl mixture of internal standard work solution was added. The internal work solution used contained 11-deoxycortisol[1]13C3 (14,3 nmol/L), corticosterone-d4 (28,5 nmol/L), 21-deoxycortisol-d4 (14,3 nmol/L), and 11-deoxycorticosterone[1]13C3 (9,0 nmol/L), which were diluted 25 × in 20% methanol. The samples were subsequently left for one hour at room temperature. Each sample was extracted twice in 1 ml methanol by using a vortex (2000 rpm), followed by centrifuging at 12000xg for 10 min at room temperature. The supernatant was transferred to tubes containing 200 mg of solid ZnCl2 for lipid precipitation^97^. The total volume of the combined supernatants was made to 4 ml by adding 2 ml methanol, and centrifuged at 12000xg for 10 min at 4 °C. The supernatant was dried under nitrogen gas in a water bath at 50 °C, re-suspended in 1 ml methanol, centrifuged at 12000xg for 10 min at room temperature, followed by addition of 1.8 ml water to the supernatant. This mixture was centrifuged at 12000xg for 10 min at 4 °C. The supernatant was loaded on C-18 SPE columns (SEClute™ SPE C18-Aq 500 mg/3 mL, code 5,138,775, Aurora Borealis Control BV, Schoonebeek, The Netherlands) pre-equilibrated with 3 ml of methanol, followed by 3 ml of water. After loading the supernatant, eluding the cartridge, the flow through was collected, columns were washed with 3 ml water, and then eluted with 2 ml methanol. The eluent was dried under nitrogen gas in a water bath at 50 °C and re-suspended in 80 µl methanol followed by addition of 120 µl water to make a final concentration of 40% methanol. Standards were prepared using dilution series from pre-prepared stock and ranged from 0.05 to 6.96 nmol/l cortisone. The standards were treated according to the same extraction procedure as described for fish eggs.

The samples were analysed using the Waters Acquity system ultra-performance liquid chromatography (uPLC) coupled with a cartridge of type XBridge™. In addition, samples were analysed with Waters TQ-S Xevo system tandem mass spectrometry (MS–MS).

### Statistical analysis

#### Egg mass and clutch size

Statistical analyses were done with R version 4.1.2^98^. To assess the difference in egg mass between females reproducing in large and small group we run linear mixed models (LMMs), using the package ‘lme4’ 1.1–27.1^99^. The normality assumptions of the LMM and the normal distribution of corticosteroid metabolites were confirmed with Shapiro–Wilk tests and Kolmogorov–Smirnov tests with Lilliefors correction together with a visual inspection of the quantile–quantile (Q-Q) plots of the model residuals^100^ using the packages ‘nortest’ 1.0–4^101^ and ‘afex’ 1.0–1^102^. To calculate the differences in egg mass, we used the mean egg dry weights from unfertilized clutches. We fitted an LMM which included egg mass as dependent variable and ‘group size’, ‘spawning sequence’ (i.e., spawning 1 or 2), and their interaction, and ‘female body condition’ as fixed factors. We included the identity of the breeding pair as a random factor to account for the repeated spawns by the same pairs. The interaction term did not significantly explain egg mass (Table S2) and the simplified model without the interaction had a lower AIC; therefore, we dropped the interaction term from the final model. In addition, we fitted a generalized mixed effect model (GLMM), assuming a negative binomial distribution, which included clutch size as dependent variable and ‘group size’, ‘spawning sequence’, their interaction, and ‘female body condition’ as fixed factors. ‘Breeder pair identity’ was included as random factor. The interaction term did not significantly explain the clutch size (Table S3) and the AIC of the model was similar to the simplified model without interaction, so both models are equivalent; therefore, the interaction term was dropped from the final model.

Model selection was based on the Akaike’s information criterion (AIC)^103^. If a fixed term had no significant effect on the response variable and the simplified model had a lower AIC than the model including this factor, it was dropped from the final model. We started model simplification by removing non-significant interaction terms, followed by main effects, with group size and fertilization state always being retained in the final model per default. Full initial models before simplification are shown in Tables S2–S6. Effect sizes were obtained by converting the statistical values (i.e., t and z) to the effect size statistic ‘Cohens’ d value’ using the package ‘effectsize’ version 0.7.0.5^104^.

#### Egg hormone concentration

We set a signal to noise (S/N) ratio equal or higher than 10 as cut-off to select the corticosteroid metabolites analysed further in this study. These were cortisone, cortisol like-compound (95% like cortisol), 11-deoxycortisol, and 11-deoxycorticosterone.

The corticosteroid metabolites 11-deoxycortisol, cortisol-like, and cortisone were log-transformed to achieve normality. Afterwards, the variables were scaled to unit variance using the function ‘scale.unit’, followed by a principal component analysis (PCA) using the ‘PCA’ function and the package ‘factoextra v. 1.0.7’^105^. In addition, a graphical representation of the first two principal components (PC) was constructed using the two PCs that explained most of the variance of the data set, by using the package ‘FactoMineR v. 2.4’^106^. The loadings of each individual sample for each steroid metabolite were extracted from the PCA, and linear models (LM) were done to determine the influence of group size and fertilization status on the individual scores of PC1 and PC2.

## Supplementary Information

Supplementary Information.

## Data Availability

The datasets analysed during the current study are available in the “Egg-mediated maternal effects in a cooperatively breeding cichlid fish” repository, https://figshare.com/s/e1e0b92a214f229d0a2b.
