# Supplementary material for: Egg-mediated maternal effects in a cooperatively breeding cichlid fish
Source: Sci Rep. 2023 Jun 16;13:9759. doi: 10.1038/s41598-023-35550-5 (PMC10276030; doi:10.1038/s41598-023-35550-5)
Supplement: Supplementary file 1 — Supplementary Information. [file 41598_2023_35550_MOESM1_ESM.docx]

**Egg-mediated maternal effects in a cooperatively breeding cichlid fish**

Maria Reyes-Contreras^1^, Bonnie de Vries^2^, J.C. van der Molen^3^, T. G. G. Groothuis^2^, Barbara Taborsky^1*^

*^1^Division of Behavioural Ecology, Institute of Ecology and Evolution, University of Bern, Wohlenstrasse 50A, CH-3032 Hinterkappelen, Switzerland*

*^2^The Groningen Institute for Evolutionary Life Science, University of Groningen, Nijenborgh 7, 9747 AG, The Netherlands.*

*^3^Laboratorium Bijzondere Chemie, Cluster Endocrinologie and Metabole Ziekten, University Medical Center Groningen, 9700 RB Groningen, The Netherlands.*

**Supplementary Information Figure S1-S2, Table S1-S6**

**Supplementary figures:**

| 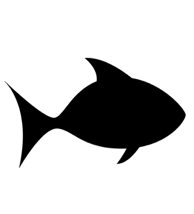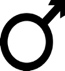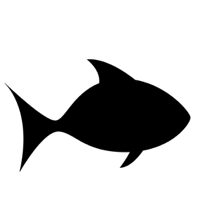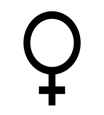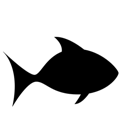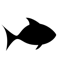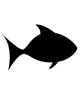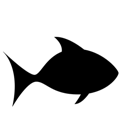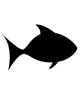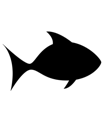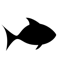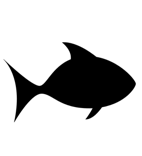 |
| --- |

**Figure S1**. Experimental set-up used to collect unfertilized clutches (‘spawning 1 and 2’). For spawning, *Neolamprologus pulcher* females and males jointly visit the breeding chamber, where the female deposits eggs on the chamber walls, which are immediately fertilized by males. In the set-up we built a “shared shelter” around a transparent divider, which could be visited simultaneously by the breeder male and the female (i.e., breeder or large helper), and where they could court each other, which stimulate females spawning. Yet, the breeders had no physical contact. This method allowed to collect unfertilized eggs from females, since the transparent divider prevented the fertilization of the eggs by the male. The breeder male (left compartment) was separated from the female (middle compartment) by a transparent partition (left grey vertical line). The rest of the group was separated from the breeders by another transparent partition (right grey vertical line; set-up adapted from^1^).


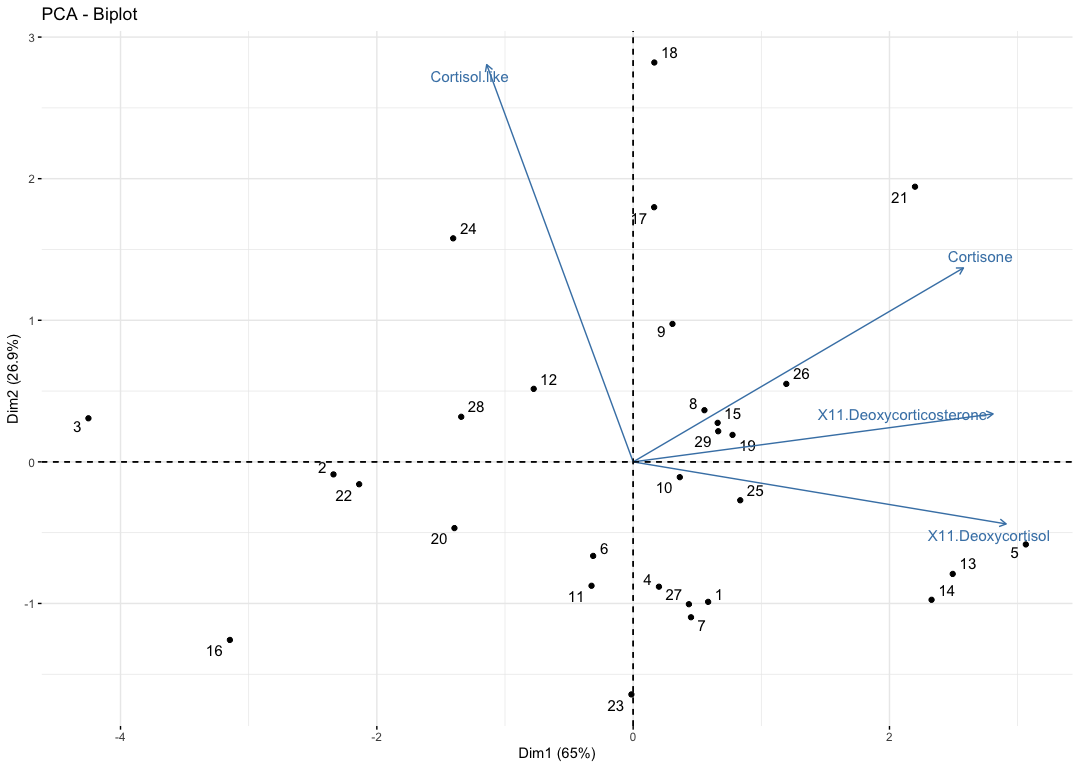


**Figure S2.** Biplot of the dimension 1 of the principal component (PC1), cortisone, 11-deoxycorticosterone, and 11-deoxycortisol load positively in this dimension whereas the cortisol-like metabolite loads in dimension 2 of the principal component (PC2).

**Supplementary Tables**

**Table S1.** Initial individual size in large groups when the groups are first established in their home tanks. The group structure was taken from Fischer et al., 2015^2^.

| Size range (cm) | Status | Number | Sex |
| --- | --- | --- | --- |
| 1.5-2.5 | Small helper | 2 | Unknown |
| 2.6-3.5 | Medium Helper | 2 | Female |
| 3.6-4.0 | Large helper | 1 | Male |
| 3.9-4.0 | Large helper | 1 | Female |
| 4.1-4.6 | Large helper | 1 | Male |
| 4.7-5.2 | Large helper | 1 | Female |
| 5.2-5.5 | Dominant breeder | 1 | Female |
| 5.5-6.0 | Dominant breeder | 1 | Male |

**Table S2**. Results of the full, initial linear mixed model (LMM), to test the effect of group size, spawning sequence and their interaction, and female body condition on egg mass. Sample sizes: small groups n = 11 clutches (spawning sequence 1: n = 7, spawning sequence 2, n = 4). Sample size: large groups n = 9 clutches, (spawning sequence 1: n = 2, spawning sequence 2: n = 7). Estimates refer to the factor levels given in brackets. Significant *p*-values are in bold (except for the intercept).

|  | Estimate ± S.E. | t | p |
| --- | --- | --- | --- |
| Intercept | 0.0002 ± 0.00009 | 2.96 | 0.014 |
| Spawning sequence (spawning 2) | 0.00009 ± 0.00006 | 1.48 | 0.17 |
| Group size (small) | 0.0000 ± 0.00006 | 0.93 | 0.37 |
| Female body condition | 0.00005 ± 0.00003 | 1.67 | 0.12 |
| Spawning sequence (spawning 2) x group size (small) | -0.00007 ± 0.00009 | - 0.74 | 0.48 |

**Table S3**. Generalized linear mixed-effect model (GLMM), to test the effect of group size, spawning sequence and their interaction, and female body condition on clutch size. Sample sizes: small groups, n = 10 clutches (spawning 1: n = 7, spawning 2: n = 3); large groups, n = 11 clutches (spawning 1: n = 4, spawning 2: n = 7). Estimates refer to factor levels given in brackets. Significant p-values are in bold except for the intercept.

|  | Estimate ± S.E. | z | p |
| --- | --- | --- | --- |
| Intercept | 2.292 ± 0.558 | 4.11 | < 0.001 |
| Spawning sequence (spawning 2) | 0.804 ± 0.224 | 3.59 | **0.00034** |
| Group size (small) | 0.565 ± 0.215 | 2.63 | **0.0086** |
| Female body condition | 0.513 ± 0.168 | 3.05 | **0.0023** |
| Spawning sequence (spawning 2) x group size (small) | -0.487 ± 0.319 | -1.53 | 0.13 |

**Table S4**. Percentage of explained variance for each dimension of the PCA on egg corticosteroids for the full data set including fertilized and unfertilized eggs from large and small groups.

|  | Eigenvalue | Variance (%) | Cumulative variance (%) |
| --- | --- | --- | --- |
| Dimension 1 | 2.60 | 64.96 | 64.96 |
| Dimension 2 | 1.08 | 26.87 | 91.84 |
| Dimension 3 | 0.22 | 5.55 | 97.38 |
| Dimension 4 | 0.11 | 2.62 | 100 |

**Table S5**. Summary table of the full initial linear model (LM), of the individual scores along PC1 investigating the effect of group size, fertilization state and their interaction on corticosteroid content in eggs. Reference categories for the estimates are given in brackets.

|  | Estimate ± S.E | t | p |
| --- | --- | --- | --- |
| Intercept | -0.791 ± 0.807 | -0.98 | 0.336 |
| Group size (small) | 0.272 ± 0.969 | 0.28 | 0.78 |
| Fertilization state (unfertilized) | 0.971 ± 0.988 | 0.98 | 0.34 |
| Group size (small) x fertilization state (unfertilized) | 0.348 ± 1.261 | 0.28 | 0.79 |

**Table S6.** Summary table of the full, initial LM, of the individual scores along PC2 of the PCA investigating the effect of group size, fertilization state and their interaction on corticosteroid metabolites content in eggs. Reference categories for the estimates are given in brackets.

|  | Estimate ± S.E | t | p |
| --- | --- | --- | --- |
| Intercept | -0.315 ± 0.538 | -0.59 | 0.56 |
| Group size (small) | 0.193 ± 0.646 | 0.3 | 0.77 |
| Fertilization state (unfertilized) | 0.753 ± 0.659 | 1.14 | 0.26 |
| Group size (small) x fertilization state (unfertilized) | -0.775 ± 0.841 | -0.92 | 0.33 |

References

1. Maldonado, M. Mate choice in a cooperative breeder. Master Thesis, University of Bern (2017).

2. Fischer, S., Bessert-Nettelbeck, M., Kotrschal, A. & Taborsky, B. Rearing-group size determines social competence and brain structure in a cooperatively breeding cichlid. *American Naturalist* **186**, 123–140 (2015).
